# Handle with care: challenges associated with ultra-processed foods research

**DOI:** 10.1093/ije/dyae106

**Published:** 2024-08-27

**Authors:** Lauren E O’Connor, Kirsten A Herrick, Keren Papier

**Affiliations:** Beltsville Human Nutrition Research Center, Agricultural Research Service, United States Department of Agriculture, Beltsville, MD, USA; Division of Cancer Control and Population Sciences, Risk Factors Assessment Branch, National Cancer Institute, National Institutes of Health, Rockville, MD, USA; Division of Cancer Control and Population Sciences, Risk Factors Assessment Branch, National Cancer Institute, National Institutes of Health, Rockville, MD, USA; Cancer Epidemiology Unit, Nuffield Department of Population Health, University of Oxford, Oxford, UK

**Keywords:** ultra-processed, health, research, nutrition

## Introduction

The World Health Organization and a number of countries have long recommended increasing the consumption of fibre, fruit and vegetables, and limiting intakes of foods that are energy dense and high in saturated fat, free sugars and salt, for disease prevention. More recently, there has been debate about whether dietary guidance about ultra-processed foods (UPFs) is needed. Ultra-processed foods are defined as ‘industrially manufactured products made up of several ingredients (formulations) including sugar, oils, fat, and salt (generally in combination and in higher amounts than in processed foods) and food substances of no or rare culinary use’.^1^ High intake of UPFs is associated with a higher risk of several poor health outcomes in observational epidemiological analyses^2^ and with excess energy intake and weight gain in one randomised controlled trial.^3^ Beyond the nutrient composition there are additional proposed mechanisms that suggest negative health outcomes from consuming high amounts of these products.^4^ For example, mechanisms that affect food choice (including e.g. cost, shelf-life, packaging, hyper-palatability and hunger stimulation/fullness suppression), mechanisms that may lead to over-consumption (e.g. including digestive processes that have yet to be described, by altering oral processing effort, eating rate, gastric emptying time, gastrointestinal transit time and perturbations in the microbiome), and mechanisms relating high consumption with disease outcomes (e.g. excess energy intake and weight gain). However, data on many of these proposed mechanisms are not supportive of these hypotheses or are lacking.^4^ There could be yet unknown pathways to consider as well. Aside from mechanisms, questions remain about whether classifying foods based on processing contributes additional value beyond conventional dietary recommendations.^5^ Further, there are noted concerns about the definition, validity and reproducibility of food processing classification systems.^6^^,^^7^ This presents challenges for interpreting associations with health outcomes observed in the literature.

**Table 1.** Description of how additives are considered across categories from the Nova food processing classification system

Nova group	Additive explanation^a^
Group 1: Unprocessed or minimally processed foods	‘Additives are usually not necessary and only exceptionally found in minimally processed foods’. ‘…Foods with vitamins and minerals added generally to replace nutrients lost during processing, such as wheat or corn flour fortified with iron and folic acid’.
Group 2: Processed culinary ingredients	‘Additives are usually not necessary and only exceptionally found in processed culinary ingredients’. ‘Also, products consisting of two Group 2 items, such as salted butter, and Group 2 items with added vitamins or minerals, such as iodised salt’.
Group 3: Processed foods	‘Processed foods often contain additives that prolong product duration, protect original properties or prevent proliferation of microorganisms (such as preservatives and antioxidants), but not additives with cosmetic functions’.
Group 4: Ultra-processed foods	‘…Application of additives including those whose function is to make the final product palatable or hyper-palatable such as flavours, colourants, non-sugar sweeteners and emulsifiers; and sophisticated packaging, usually with synthetic materials’. ‘…Additives with cosmetic functions (flavours, flavour enhancers, colours, emulsifiers, emulsifying salts, sweeteners, thickeners and anti-foaming, bulking, carbonating, foaming, gelling and glazing agents) in their list of ingredients’.

a Additive explanation from Martinez-Steele *et al., Nature Food* 2023.

So what is an epidemiologist to do? The objective of this commentary is to discuss methodological considerations for researchers to be mindful of, when conducting or interpreting research on the topic of ultra-processed foods and human health outcomes, particularly for observational study designs using self-reported dietary data. This is not meant to be an exhaustive review of the literature on this topic.

## Challenges in understanding food formulation and food processing

There is question whether Nova (a four-group classification framework of processed foods) is classifying foods based on formulation or processing, which is compounded by defining UPFs as products ‘made of several ingredients (formulations)’ as noted above.^1^ Formulation is defined as the combination of ingredients and additives added and prepared according to prescribed methods, to produce a product intended for further processing or ready for consumption.^5^ Food processing is ‘the use of methods and techniques involving equipment, energy and tools to transform agricultural products such as grains, meats, vegetables, fruits and milk into food ingredients or finished food products’.^8^ Formulation and processing are two different aspects of food production. It is likely that application of Nova to dietary data is classifying foods based on formulation rather than processing, based on the use of ingredient lists for classification, as described below in more detail. Information on the science of food processing and concerns about Nova from the food science community are described elsewhere.^1^

## Challenges with dietary assessment

Dietary assessment has well-known limitations that affect many aspects of nutritional epidemiology.^9^ We highlight here additional layers that researchers should consider when assessing relationships between UPF intake and health. To assess intake of ultra-processed foods using the Nova system, researchers are first tasked to identify whether the food is ‘industrially manufactured’ or a ‘culinary preparation’,^1^ during data collection. In other words, researchers must collect data on whether a self-reported food was prepared/prepackaged vs handmade/home-made. This is generally not possible with food frequency questionnaires (FFQs), because FFQs assess broad groups of foods and beverages, thus individual food items are not recorded. Researchers then assign all food items captured by each FFQ question to one Nova category, though most would likely include products that fall along the Nova spectrum. Other dietary assessment tools, such as 24-h dietary recalls or records, ask respondents to report each item consumed within a 24-h period. This provides a list of all items consumed, with detail on the item level, to help inform whether it was ‘industrially manufactured’ or a ‘culinary preparation’. If a brand name is reported, for example, for a particular brand of spaghetti sauce, then it can easily be identified as an ‘industrially manufactured’ product which can then undergo the classification steps described by Martinez-Steele *et al.*^1^ using the ingredient list on the package or label.

However, most dietary assessment tools have limited ability to collect brand name data, in part because of respondents’ limited ability to recall brand names accurately. Another related limitation is the restricted amount of branded foods available in the underlying databases. Challenges remain even if a dietary assessment tool records whether a food item, e.g. tomato sauce, is purchased or home-made, due to a lack of consensus or understanding on what ‘home-made’ means to each respondent.^10^ Respondent 1 may consider ‘tomato sauce’ as home-made if it was made at home in their kitchen and they add onions and fresh basil to a jarred pre-packaged sauce. Respondent 2 may consider ‘tomato sauce’ as home-made only if fresh tomatoes, onions and basil are simmered for hours on the stove. To probe this information from respondents, to obtain the needed level of detail for accurate Nova classification using current dietary assessment technology on every item reported by the respondent, would add insurmountable time and burden on the participant, jeopardising validity and totality of the reported data due to fatigue. Therefore, methodological innovation is needed to improve assessments of UPF intakes, while also not overburdening research volunteers.

## Challenges with underlying dietary databases

A large proportion of publications about health associations of UPFs are from previously conducted observational cohort studies that use FFQs or 24-h recall tools that pre-date Nova. It is not possible to retroactively probe respondents about home-made dishes, brand names etc. on dietary datasets that are already collected. In these scenarios, assumptions need to be made (and should be documented) to establish whether something is ‘industrially manufactured’ or a ‘culinary preparation’ for further Nova classification. This is generally determined by the description of food codes provided in the underlying dietary database that provides the food and nutrient information. Searching for terms such as ‘ready-to-eat’ or ‘frozen’ (as in a frozen pizza) can be indicators that an item may be ‘industrially manufactured’.^11^ Once an item is labelled as ‘industrially manufactured’, the next step is to then determine if the formulation or list of ingredients contains any of the hallmark characteristics of an ultra-processed food,^1^ described in more detail below. However, these ingredients are generally not provided in dietary databases, and product formulations change over time, often outpacing updates in dietary databases.

## Challenges with using additives for classification

A key defining feature of a UPF is the presence of certain ingredients or additives, such as high-fructose corn syrup, artificial sweeteners, additives with ‘cosmetic functions’, modified starches, emulsifiers, thickeners and many more.^1^ If researchers have access to food labels for their dietary data (e.g. if designing a menu for a controlled feeding study or dietary intervention, or using grocery purchase data), it is possible to view the list of ingredients of each product for these hallmark UPF characteristics. However, according to the Nova guidelines, the function of the additive needs to be taken into consideration for accurate classification.^1^Table 1 outlines the guidance about additives for Nova classification. The Nova system recognises that some additives are essential for food safety and nutrition security, and thus should not be the reason that a food or beverage be labelled as ultra-processed. For example, the process of enrichment (adding back nutrients that were lost during processing^1^) or the use of preservatives that prolong product duration or prevent microbial growth does not make a food ultra-processed. Additives that have a ‘cosmetic function’ to improve palatability are the hallmarks that demarcate a food as ultra-processed. However, additives often serve multiple purposes. Sodium additives, for example, are the most ubiquitous and oldest preservatives in any food supply. The function of sodium additives may be for preservation as well as for taste and flavour enhancements. Another example is ascorbic acid which is used for vitamin C fortification but is also used to avoid browning of fruit to maintain quality, appearance and acceptability. It is unclear how a researcher can be sure what the purpose of each additive is in a particular food, to determine whether the function of that additive delineates each individual food or beverage as processed or ultra-processed. Extensive knowledge of food science and regulatory science are needed for accurate classification.

## Challenges with reliability and validity of nova

Information on the inter-rater reliability of applying the Nova system to dietary intake data has been published, but is inconsistent, with some studies finding moderate to high assessor agreement and others finding low agreement.^12^ There are also challenges to validating the Nova food processing system as has been done for other diet quality metrics (e.g. the Health Eating Index).^13^ In other words, how does one know if the foods in Group 4 undergo more steps or intensity of processing than foods in Group 3? Food processing techniques and unit operations (i.e. steps in the process) are extremely complex variables across different food products,^14^ and are most likely proprietary information held by the manufacturer. Further, questions remain as to whether Nova is based on processing, as described above, or if the system rather identifies formulation (in fact, ‘formulation of ingredients’ is the basis of the UPF definition^1^) or a combination of the two.^5^

Objective biomarkers are often proposed to improve or validate self-reported dietary data by reducing measurement error.^15^ There have been efforts to identify potential biomarkers of ultra-processed food intake via metabolomics. For example, increases in benzoate metabolism were higher after consumption of an ultra-processed dietary pattern compared with an unprocessed dietary pattern in a crossover, domiciled, randomised, controlled feeding trial of 20 participants.^16^ This may be reflective of sodium benzoates which are widely used FDA-approved food additives in the US food supply, most often used in beverages and condiments. Metabolites related to intake of artificial sweeteners were also higher after the ultra-processed vs unprocessed dietary patterns,^16^ and artificial sweeteners are a hallmark characteristic of UPFs,^1^ again largely used in beverages. However, reliance on biomarkers such as these may underestimate intake of UPFs because it is not representative of the wide array of foods in this group, and it is unclear how that affects measurement error correction methods such as regression calibration.

## Considerations for variation across food supplies

It is critical to consider how food supplies, food processing practices and food preparation habits differ across countries, and thus how classifying foods based on processing would affect public health in different ways depending on the composition and regulation of the food supply. Over 50% of energy intake in the USA^17^ and in the UK^12^ comes from UPFs. In contrast, countries such as Italy or Brazil have as little as 20% of total energy intake from UPFs.^18^^,^^19^ Most UPFs consumed in Brazil are foods that nutritionists and public health professionals would agree are discretionary food items (e.g. cakes, pies and cookies; fast food dishes; sugar-sweetened beverages etc.).^19^ Therefore, recommendations to reduce UPFs may be a reasonable public health tool to improve dietary intake of individuals living in Brazil. However, in the USA and the UK, the group of UPFs becomes much broader and more heterogeneous. Discretionary items, such as sugar-sweetened beverages, packaged chips and candy are often grouped with items that can contribute positively to diet quality, for example breakfast cereals, whole-meal bread and soya milk, as they are enriched with nutrients often lacking in the diet (i.e. vitamins A, C, D and E, calcium, iron, magnesium). Breakfast cereals and bread make up around a quarter of the energy intake from UPFs for adults and children in the USA^17^^,^^20^ and the UK.^21^ These foods, among other voluntarily fortified foods, contribute meaningfully to micronutrient intake for US^22^ and UK populations.^23^ Though the act of fortification is not the reason these foods are ultra-processed (see Table 1), they are often guilty by association, as other additives are used in production of these products. Food fortification success stories in the USA and the UK pre-date the boom of processed and ultra-processed food consumption, when people in the USA or the UK prepared and consumed most of their meals at home.^24^^,^^25^ It is difficult to meet all nutrient needs in countries like the USA and UK without consumption of processed or ultra-processed foods. This is exemplified in the food pattern modelling that is used to develop the recommended dietary patterns from the USA and UK dietary guidelines.^26^^,^^27^ These modelling approaches include fortified foods to meet nutrient targets that are still not met for all populations (e.g. vitamin D and iron for women). Recommendations to avoid fortified foods that are considered UPFs may put those recommended dietary patterns even further from the nutrient targets of the population.

## Available resources and future directions

This paper outlines challenges of applying the Nova food classification system to dietary data for researchers to consider when interpreting their findings or designing future studies. There are continuous advancements in dietary assessment tools and standardised codification of dietary databases for improved Nova classification. For example, dietary assessment tools are being developed to specifically measure intake of UPFs according to Nova.^28^ Further, standardised Nova classification for all foods and beverages for all cycles of the USA’s National Health and Nutrition Examination Survey is available, and a detailed description of the method was recently published to increase transparency.^11^ This method can be translated to other dietary databases or dietary assessment tools that link to the United States Department of Agriculture’s dietary databases. For example, this method has been translated to codify all foods and beverages available for report in the US National Cancer Institute’s Automated Self-Administered 24-Hour (ASA24) Dietary Assessment Tool. It should be noted that this method has not been compared against food labels, because many of the food codes in the underlying US dietary databases are not linked directly to a brand name or a food label. Therefore validity is still a concern, but at least the assumptions are standardised and thus results would be comparable across studies. There needs to be more research to quantify the measurement error resulting from the noted methodological limitations, whether the measurement error is concerning enough to change the rank order of intake in a population, and how that affects associations with health outcomes. Though improvements in dietary assessment methods would better advance our understanding on this topic, it is important to consider what level of measurement error we are comfortable with and when the methods are sufficient.

## Conclusions

Decades of research have helped refine public health recommendations and dietary guidelines. To achieve a similar understanding of the impact of UPFs on health, and potentially inform well-established dietary recommendations, more robust research is needed to clarify what features of foods (e.g. processing, additives, formulation) constitute the label ‘ultra-processed’ and to address several outstanding challenges related to assessment and operationalisation of UPF intake. There are common misconceptions and misunderstandings about food formulation and food processing by the nutrition research community. Thus, it is essential to consult or collaborate with experts in food science when conducting research on UPFs and health, to ensure that the complex field of food processing is accurately described and investigated. The research community can recognise and address these challenges in their research while seeking to advance the field of UPFs consumption and health.

## Disclaimer

The opinions expressed by the authors are their own and this material should not be interpreted as representing the official viewpoint of the U.S. Department of Agriculture, the U.S. Department of Health and Human Services, the National Institutes of Health or the National Cancer Institute. For the purpose of open access, the author(s) has applied a Creative Commons Attribution (CC BY) licence to any Author Accepted Manuscript.
