# Correction to: Handle with care: challenges associated with ultra-processed foods research

**DOI:** 10.1093/ije/dyae133

**Published:** 2024-10-08

This is a correction to: Lauren E O’Connor, Kirsten A Herrick, Keren Papier, Handle with care: challenges associated with ultra-processed foods research, International Journal of Epidemiology, Volume 53, Issue 5, October 2024, dyae106, https://doi.org/10.1093/ije/dyae106

In the version originally published, the second author was incorrectly associated with the affiliation:

Beltsville Human Nutrition Research Center, Agricultural Research Service, United States Department of Agriculture, Beltsville, MD, USA

The article has been updated to remove this association.

Additionally, in the section ‘Challenges with using additives for classification’ a reference to acetic acid has been corrected to read ‘ascorbic acid’.

